# Anticoagulation Treatment in Patients with Septic Thrombophlebitis of the Internal Jugular Vein

**DOI:** 10.5811/westjem.47130

**Published:** 2025-11-26

**Authors:** Atsushi Senda, Kiyohide Fushimi, Koji Morishita

**Affiliations:** * Institute of Science Tokyo, Graduate School of Medical and Dental Sciences, Department of Acute Critical Care and Disaster Medicine, Tokyo, Japan; † Toda Chuo General Hospital, Department of Emergency Medicine, Toda, Saitama, Japan; ‡ Institute of Science Tokyo, Graduate School of Medical and Dental Sciences, Department of Health Policy and Informatics, Tokyo, Japan

## Abstract

**Introduction:**

Septic thrombophlebitis of the internal jugular vein (STIJV), or Lemierre syndrome, is a rare, life-threatening condition. Anticoagulant use for managing STIJV remains unclear due to ambiguous diagnostic criteria and a lack of robust evidence. We evaluated the clinical benefits and risks of anticoagulants in patients with STIJV.

**Methods:**

In this retrospective study we used data from over 1,700 hospitals, retrieved from a nationwide Japanese database. We used multivariate logistic regression and propensity score matching to adjust for confounding variables (age, sex, Charlson Comorbidity Index, level of consciousness, use of mechanical ventilation, use of disseminated intravascular coagulation, admission to intensive care unit, history of diabetes, use of noradrenaline, diagnosis of acute renal failure, and diagnosis of cerebral infarction). We also conducted instrumental variable estimation to account for the impact of unmeasured covariates. The primary outcome was in-hospital mortality; the secondary outcomes were 90-day mortality, major bleeding events, and length of stay (LOS) in hospital.

**Results:**

Among the 523 patients diagnosed with STIJV between April 1, 2014–March 31, 2022, 343 (65.6%) were excluded due to lack of appropriate treatment initiation for STIJV. Overall, 180 patients (34.4%) met the inclusion criteria; the data of 156 patients (31.1%) were ultimately analysed. Of these, 86 (55.1%) received anticoagulants, which neither significantly improved nor worsened survival outcomes. The in-hospital mortality was 3.39% and 1.69% and 90-day mortality was 2.54% and 1.69%, respectively, in patients who did and did not receive therapy, (*P* = .56 and .99, respectively). The adjusted odds ratio (AOR) for in-hospital and 90-day mortality was 0.858 (95% CI, 0.126–5.826, *P* = .88) and .991 (95% CI, .932–1.055, *P* = .79), respectively. The LOS was longer in those receiving anticoagulants (mean, 29.2 vs 21.8 days, AOR 11.7 days longer, 95% CI, 4.11–19.20, *P* < .01), potentially due to dose adjustment or clinical decision-making. Subgroup analysis comparing unfractionated heparin and direct Xa inhibitors showed similar in-hospital mortality outcomes: 4.54% in the unfractionated heparin group (AOR 2.361, 95% CI, 0.32–17.40; *P* = .40) and 3.03% in the direct Xa inhibitor group (AOR 0.444, 95% CI, 0.032–6.23; *P* = .55), respectively.

**Conclusion:**

In the largest study of septic thrombophlebitis of the internal jugular vein to date, we found that early initiation of anticoagulation treatment was not statistically associated with survival. Therefore, anticoagulant use should be determined based on individual patient characteristics. Further research is warranted to improve the quality of evidence for this rare disease.

## INTRODUCTION

Septic thrombophlebitis of the internal jugular vein (STIJV), or Lemierre syndrome, is a rare but life-threatening condition.^1^ It typically follows an oropharyngeal infection, leading to local invasion of the pharyngeal space and IJV, resulting in septic thrombophlebitis within one to three weeks.^2^,^3^ Despite its severity, STIJV management and outcomes remain poorly understood owing to its rarity^4^ and inconsistent diagnostic criteria.^5^ Some studies define STIJV based on the presence of *Fusobacterium necrophorum*, regardless of symptoms,^6^,^7^ while others base it on septic emboli findings, irrespective of the pathogen.^3^,^8^

These inconsistencies complicate clinical decision-making. Clinicians often face challenges when the causative pathogen is unclear and evidence-based guidance is lacking.^9^ A long-debated issue^7^,^9^–^11^ is whether anticoagulation treatment should be initiated. Although this therapy is frequently used,^9^,^12^ its efficacy and safety lack robust evidence.^13^,^14^ In this study, we aimed to evaluate the clinical benefits and risks of anticoagulation therapy in patients with STIJV, regardless of the causative pathogen. This study provides clinicians with evidence-based tools to manage this challenging condition.

## METHODS

### Study Design

In this retrospective study we assessed whether anticoagulation therapy could confer a survival advantage. We used multivariate logistic regression and propensity score matching (PSM) to adjust for intergroup baseline differences. The primary outcome was in-hospital mortality, while the secondary outcomes were 90-day mortality, major bleeding events during hospitalization, and hospital length of stay (LOS). This study complied with all 12 quality improvement strategies proposed by Worster et al.^15^ The results were reported according to the Strengthening the Reporting of Observational studies in Epidemiology (STROBE) guidelines (Appendix 1).

### Ethics Approval and Consent to Participate

The study adhered to the principles of the 1975 Declaration of Helsinki and its subsequent revisions. Ethical approval was granted by the institutional review board of our institution (#788; April 2020). The requirement for informed consent was waived owing to the retrospective nature of the study and use of anonymous patient and hospital data.

### Data Resources

We extracted data from the Japanese Diagnosis Procedure Combination (DPC) system, a nationwide case-mixed patient classification framework designed to standardize electronic claims processing and enhance transparency in hospital performance. Over 1,700 hospitals, including 82 academic institutions, participate in the system, which also streamlines electronic payment systems and fosters accountability in healthcare operations. The database captures the following elements: 1) major diagnosis categories with detailed diagnostic groupings; 2) hospitalization details, including the location and method of admission; 3) patient demographics; 4) surgical procedures performed; 5) medications and other adjuvant therapies administered; and 6) associated comorbidities and complications.^16^–^18^

**Population Health Research Capsule**

What do we already know about this issue?

*Septic thrombophlebitis of the internal jugular vein (STIJV) is a rare but severe infection. Despite the use of anticoagulants their benefit remains uncertain.*

What was the research question?

*Does anticoagulation improve mortality or clinical outcomes in patients with STIJV?*

What was the major finding of the study?

*Anticoagulation use was not statistically association with survival (OR 0.858; 95% CI, 0.126–5.826; P= .88); hospital stay was 11.7 days longer with anticoagulants (P < .01).*

How does this improve population health?

*Evidence supports tailored use of anticoagulants in STIJV, helping reduce harm and resource use.*

### Study Population

In this study we registered hospitalized patients diagnosed with STIJV between 1 April 1, 2014–March 31, 2022. To ensure the accuracy of the diagnostic labels, we included only patients who showed evidence of appropriate intervention at the time of diagnosis. Specifically, we excluded individuals without records of submitted blood cultures or without antibiotic treatment initiated on the day of diagnosis. The exclusion criteria were as follows: 1) incomplete data for any variable included in the analysis; 2) < 16 years of age; 3) pregnancy; and 4) discharge within 48 hours of admission. We applied the last criterion to address the immortal time bias because disease severity was assessed based on the treatment intensity provided during this period.

### Data Collection

The selection criteria and variables required for the analysis were provided to an abstractor specializing in database management, who was blinded to the study hypothesis. Data collection was carried out using abstraction forms. We confirmed the reliability of the process by achieving perfect interrater agreement. The collected covariates included age, sex, consciousness level at admission (alert, comatose, or other), Charlson Comorbidity Index (CCI), concurrent diagnosis at admission, hospitalization location, and use of mechanical ventilation and vasopressors. We also collected data on post-admission complications, LOS, and discharge status (alive or deceased) to evaluate patient prognosis.

### Patient Groups and Treatment Protocols

The anticoagulant treatment group included patients who had received warfarin, unfractionated heparin, dalteparin, edoxaban, rivaroxaban, apixaban, or fondaparinux at admission. Patients who survived to discharge were included in the survival group. Patient severity was adjusted using multivariable logistic regression and PSM with the following covariates: age, sex, CCI, level of consciousness, use of mechanical ventilation, use of disseminated intravascular coagulation, admission to intensive care unit (ICU), history of diabetes, use of noradrenaline, diagnosis of acute renal failure, and diagnosis of cerebral infarction.

### Statistical Analysis

We performed severity adjustment between patients with STIJV who received anticoagulation treatment and those who did not using a multivariate logistic regression analysis. The defined covariates were included in a generalized linear model. We categorized the population into two groups in a 70/30 ratio, with 70% defining the parameters of the regression model and the remaining data assessing its performance. We assessed the model accuracy using the area under the receiver operating characteristic curve and conducted the Hosmer-Lemeshow test to evaluate its goodness of fit of the constructed model.

To ensure the robustness of our results, we performed PSM under the following conditions: 1:1 K-nearest neighbor matching without replacement, matching caliper set at 0.2 times the standard deviation of the logit-transformed propensity score. Luo et al previously reported on the details on the PSM method.^19^ Additionally, we performed instrumental variable estimation to further evaluate the robustness of the results, using institution identification numbers as the instrument. The Durbin-Wu-Hausman test was subsequently applied to assess the endogeneity of this variable. We applied the chi-square test to compare outcomes between the PSM groups. Statistical analyses were conducted using R software v4.4.2 (The R Foundation for Statistical Computing, Vienna, Austria).

## RESULTS

**Figure 1:**
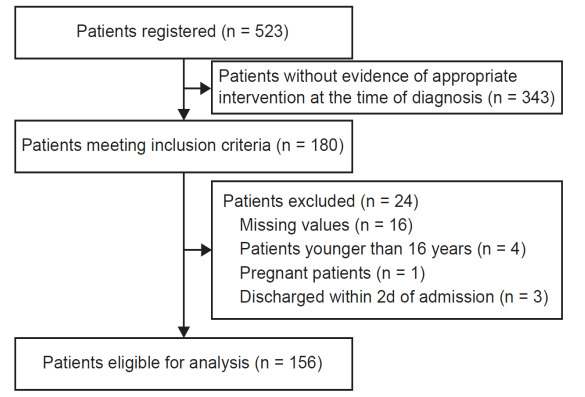
Flowchart depicting patient selection in a study of anticoagulation in patients with septic internal jugular thrombophlebitis.

The patient selection process is shown in Figure 1. Among the 523 patients diagnosed with STIJV during the study period, 180 met the inclusion criteria. A total of 156 patients were included in the analysis. We excluded 24 patients due to missing values (n = 16), age < 16 years (n = 4), pregnancy (n = 1), and discharge within two days of admission (n = 3).

Hospitalized patients diagnosed with STIJV between April 1, 2014–March 31, 2022 were registered in this study based on data extracted from the Japanese DPC system. The anticoagulants administered to the treated group were as follows: unfractionated heparin (n = 66, 42.3%); warfarin (n = 31, 19.9%); apixaban (n = 19, 12.2%); edoxaban (n = 5, 3.2%); rivaroxaban (n = 5; 3.2%); dalteparin (n = 2, 1.3%); and fondaparinux (n = 2, 1.3%). The patient demographics are presented in Table 1, showing that patients treated with anticoagulants had higher scores on the CCI (median of 0 vs 1 in patients not treated with anticoagulants), a higher rate of coma (2% vs 0% in patients not treated with anticoagulants), mechanical ventilation (2% vs 0% in patients not treated with anticoagulants), and administration of noradrenaline (12.8% vs 1.4% in patients not treated with anticoagulants).

Patients treated with anticoagulants received the following agents: unfractionated heparin (n = 66, 42.3%); warfarin (n = 31, 19.9%); apixaban (n = 19, 12.2%); edoxaban (n = 5, 3.2%); rivaroxaban (n = 5, 3.2%); dalteparin (n = 2, 1.3%); and fondaparinux (n = 2, 1.3%).

**Figure 2:**
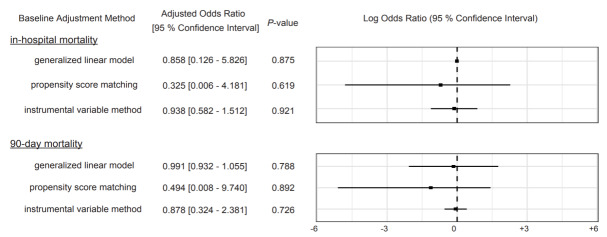
Forest plot comparing in-hospital mortality and 90-day mortality between patients treated with anticoagulants and those not treated with anticoagulants in a study of anticoagulation in patients with septic internal jugular thrombophlebitis.

The performance of the established generalized linear model was robust, with an area under the receiver operating characteristic curve of 0.92 (Supplementary Figure 1) and well calibrated; Hosmer-Lemeshow goodness of fit, *P* =.89 (Supplementary Figure 2). The results of the main analysis are shown in Figure 2. Using this model, the adjusted odds ratio (AOR) for in-hospital mortality was estimated to be 0.858 (95% CI, 0.126–5.826, *P* = .88). Similarly, the AOR for 90-day mortality was 0.991 (95% CI, 0.932–1.055, *P* = .79), indicating that anticoagulation treatment neither significantly improved nor worsened the survival rates.

We estimated AORs for in-hospital mortality and 90-day mortality between patients treated with anticoagulants and those not treated with anticoagulants using a generalized linear model and propensity score matching, respectively. Baseline characteristics after 1:1 PSM are presented in Table 2. Following PSM, the estimated OR for in-hospital mortality was 0.325 (95% CI, 0.006–4.181, *P* = .62). Similarly, the AOR for 90-day mortality was 0.494 (95% CI, 0.008–9.740, *P* = .62), consistent with the results of the main analysis. The result of Durbin-Wu-Hausman test was *P* =.328e–12, indicating endogeneity of the instrumental variable we enveloped. The estimated OR for in-hospital mortality was 0.938 (95% CI, 0.582–1.512, *P* = .92), and the AOR for 90-day mortality was 0.878 (95% CI, 0.324–2.381, *P* = .73), which further supports the robustness of the study.

**Figure 3:**
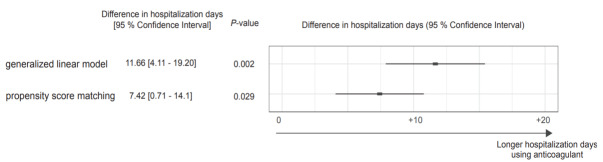
Forest plot comparing the length of hospitalization between patients treated with anticoagulants and those not treated with anticoagulants in a study of anticoagulation in patients with septic internal jugular thrombophlebitis.

**Figure 4:**
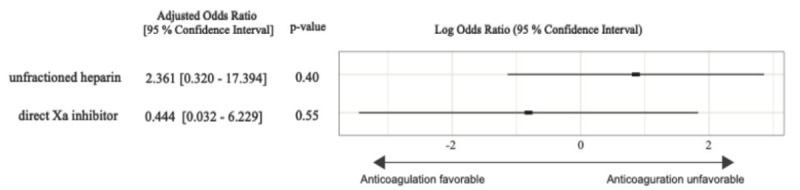
Forest plot comparing in-hospital mortality between patients treated with unfractionated heparin or the direct Xa inhibitor and patients not treated with any anticoagulants in a study of anticoagulation in patients with septic internal jugular thrombophlebitis. Analysis was performed using a generalized linear model.

In the group treated with anticoagulants, one patient (1.7%) experienced gastrointestinal bleeding during hospitalization; however, the patient survived without requiring additional intervention. Figure 3 illustrates the duration of hospitalization for both groups, showing longer LOS among patients receiving anticoagulants in both analyses. Subcategorical analysis, estimating the treatment effects of unfractionated heparin and the direct Xa inhibitor, is shown in Figure 4. The estimated OR for in-hospital mortality was 2.361 (95% CI, 0.320–17.394, *P* = .40) in the patient treated with unfractionated heparin and 0.444 (95% CI, 0.032–6.229, *P* = .55) in the patient treated with direct Xa inhibitor compared with the patient who received none of the anticoagulants.

We estimated differences in the length of hospitalization between patients treated with anticoagulants and those not treated with anticoagulants using a generalized linear model and PSM, respectively.

## DISCUSSION

In this largest study of STIVJ to date, no survival benefit from anticoagulation therapy was found in patients with STIJV; however, an association between anticoagulation therapy and prolonged hospitalization was noted. Clinicians often face major challenges when treating patients with Lemierre syndrome, or STIJV, owing to limited information. Whether anticoagulation treatment should be initiated in these patients has been debated; however, no systematic study of STIJV has been performed due to the rarity of this condition. To the best of our knowledge, this is the first study to assess the effectiveness of anticoagulants in this specific patient group.

This study showed no survival benefit from anticoagulation therapy in patients with STIJV. However, it showed an association between anticoagulation therapy and prolonged hospitalization. Clinicians often face major challenges when treating patients with Lemierre syndrome owing to limited information. Whether anticoagulation treatment should be initiated in these patients has been debated; however, to the best of our knowledge, this is the first study to assess the effectiveness of anticoagulants in this specific patient group.

Several studies have investigated this condition under the term Lemierre syndrome or disease. However, we focused on patients with STIJV. This approach was chosen because the concept of Lemierre syndrome is abstract; the definition of study populations has varied widely across previous studies. Notably, some studies included patients wherein *F necrophorum* was detected, regardless of clinical presentation.^7^,^20^ By contrast, our study included patients with STIJV, irrespective of the pathogen. This inclusion criterion was justified, as clinical decisions were often made before the availability of bacteriological results.

Successful cases have been reported both with^21^–^25^ and without anticoagulation therapy.^26^–^30^ A study analyzing 712 cases of Lemierre syndrome suggested that anticoagulation therapy may represent a favorable prognostic factor.^9^ However, this analysis was based on reported cases, introducing potential publication bias and issues of pseudo-correlation, as the study period spanned 17 years, during which the use of anticoagulation therapy became more common.^5^,^9^,^12^–^14^ Despite differences in study populations, our findings align with those of a previous study showing no significant differences in outcomes between patients with jugular vein thrombosis who received therapeutic, prophylactic, or no anticoagulation therapy.^7^

In the current study, we applied three complementary adjustment methods to mitigate differences in baseline characteristics. The model demonstrated excellent predictive accuracy (AUROC = 0.92) and confirmed population balance between the anticoagulant-treated and untreated groups after PSM, thereby addressing indication bias. Furthermore, the consistency of the instrumental variable estimation results with those from the other analyses suggests that residual confounding is unlikely to have substantially affected the findings.

Using the PSM analysis, only one patient (1.7%) who received anticoagulation therapy experienced gastrointestinal bleeding during hospitalization. This finding aligns with previous findings indicating that anticoagulation therapy is relatively safe, without major bleeding complications attributed to it.^21^–^24^ However, the prolonged hospitalization observed in patients receiving anticoagulation therapy necessitates further investigation. This may reflect the challenges physicians face in dose adjustment or a preference for keeping patients hospitalized until thrombosis resolves.

Although the subgroup analysis comparing unfractionated heparin and direct Xa inhibitors showed no apparent difference in in-hospital mortality between the two anticoagulants. The estimated OR for in-hospital mortality was 0.444 for direct Xa inhibitors, compared with 2.361 for unfractionated heparin. This finding may suggest the potential superiority of direct Xa inhibitors. A larger scale study is warranted to confirm this observation.

Lemierre syndrome is more common in men than in women, primarily affecting younger patients.^31^–^33^ This tendency was also observed in our study; however, the median age in our cohort was 65 years of age (IQR 39–79 years). This trend may be attributed to Japan’s aging population and the fact that antibiotics are administered more frequently to younger individuals than older adults, as indicated by surveillance data in Japan.^34^

## LIMITATIONS

This study has some limitations. First, bacteriological results confirming the pathogen were not available, preventing further detailed analyses. Second, to address immortal time bias, we excluded patients who stayed for < 2 days from the analysis. However, only three patients were excluded, which was unlikely to have affected the results of the study. Third, to confirm the robustness of the results, we used instrumental variable estimation. Although endogeneity of the instrument was confirmed, the extent to which the findings are fully reliable remains uncertain.

Finally, owing to the unique characteristics of our dataset, we did not evaluate long-term outcomes such as local thrombosis progression, which are common measures of treatment effects in other studies. Despite these limitations, this is the only study to thoroughly assess the effect of anticoagulation treatment in patients with STIJV while carefully addressing indication bias.

## CONCLUSION

This is the largest and first study to assess the effectiveness of anticoagulants in patients with septic thrombophlebitis of the internal jugular vein. Early initiation of anticoagulation therapy was not statistically associated with survival benefit. When comparing unfractionated heparin with direct Xa inhibitors, the findings suggest a potential superiority of direct Xa inhibitors. Larger scale studies, particularly those assessing the clinical benefits of direct Xa inhibitors, are warranted to strengthen the evidence base for this rare condition.

## Supplementary Information







## Figures and Tables

**Table 1:** Characteristics of patients and hospitals investigated in a study of anticoagulation in patients with septic internal jugular thrombophlebitis.

Patient or hospital characteristic	Patient not treated with anticoagulants	Patient treated with anticoagulants	SMD
Number of participants, n	70	86	
Age (years), median [25^th^–75th percentiles]	68.50 [45.75, 79.00]	61.50 [38.25, 77.75]	0.149
Sex, female, n (%)	34 (48.6)	35 (40.7)	0.159
Charlson Comorbidity Index, median [25^th^-75th percentiles]	0 [0, 1]	1 [0, 2]	0.388
Consciousness, alert, n (%)	55 (78.6)	70 (81.4)	0.071
Consciousness, coma, n (%)	0 (0.0)	2 (2.3)	0.218
Mechanical ventilation use, n (%)	0 (0.0)	2 (2.3)	0.218
Disseminated intravascular coagulation, n (%)	5 (7.1)	7 (8.1)	0.038
Admission to ICU, n (%)	5 (7.1)	15 (17.4)	0.318
History of diabetes, n (%)	5 (7.1)	14 (16.3)	0.287
Use of noradrenaline, n (%)	1 (1.4)	11 (12.8)	0.453
Diagnosis of acute renal failure, n (%)	7 (10.0)	8 (9.3)	0.024
Diagnosis of cerebral infarction, n (%)	2 (2.9)	4 (4.7)	0.094

*ICU*, intensive care unit; *SMD*, standardized mean difference.

**Table 2:** Patient and hospital characteristics after propensity score matching in a study of anticoagulation in patients with septic internal jugular thrombophlebitis.

Patient or hospital characteristic	Patient not treated with anticoagulants	Patient treated with anticoagulants	SMD
Number of participants, N	59	59	
Age (years), median [25^th^–75th percentiles]	65 [36.5, 78]	62 [38, 78.5]	0.023
Sex, female, n (%)	25 (42.4)	24 (40.7)	0.034
Charlson Comorbidity Index, median [25^th^–75th percentiles]	0 [0,1]	0 [0,1]	0.038
Consciousness, alert, n (%)	51 (86.4)	48 (81.4)	0.139
Consciousness, coma, n (%)	0 (0.0)	0 (0.0)	<0.001
Mechanical ventilation use, n (%)	0 (0.0)	0 (0.0)	<0.001
Disseminated intravascular coagulation, n (%)	4 (6.8)	4 (6.8)	<0.001
Admission to ICU, n (%)	5 (8.5)	4 (6.8)	0.064
History of diabetes, n (%)	5 (8.5)	3 (5.1)	0.135
Use of noradrenaline, n (%)	1 (1.7)	1 (1.7)	<0.001
Diagnosis of acute renal failure, n (%)	5 (8.5)	4 (6.8)	0.064
Diagnosis of cerebral infarction, n (%)	0 (0.0)	0 (0.0)	<0.001

We performed propensity score analysis under the following conditions: 1:1 K-nearest neighbour matching without replacement, matching caliper set at 0.2 times the standard deviation of the logit-transformed propensity score.

*ICU*, intensive care unit; *SMD*, standardized mean difference.
