# Supplementary figures and images for: Anticoagulation Treatment in Patients with Septic Thrombophlebitis of the Internal Jugular Vein
Source: West J Emerg Med. 2025 Nov 26;26(6):1590–7. doi: 10.5811/westjem.47130 (PMC12698176; doi:10.5811/westjem.47130)

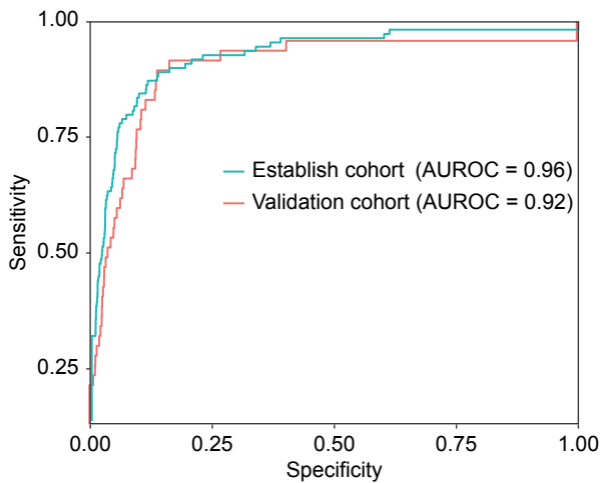

Supplement: Supplementary file 1 [file wjem-26-1590-s001.pdf]

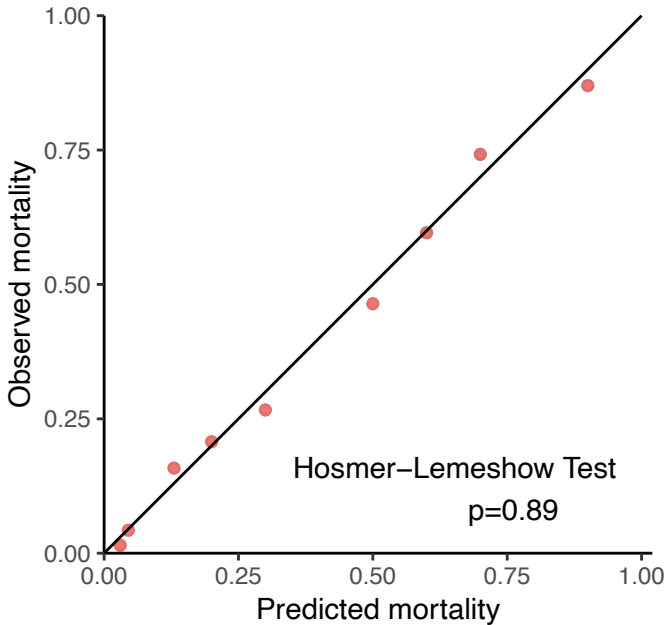

Supplement: Supplementary file 2 [file wjem-26-1590-s002.pdf]
